# The Effects of Alkaline pH on Microleakage of Mineral Trioxide Aggregate and Calcium Enriched Mixture Apical Plugs

**Published:** 2016-03

**Authors:** Hossein Mirhadi, Fariborz Moazzami, Saeed Rangani Jahromi, Sareh Safarzade

**Affiliations:** 1 Dept. of Endodontics, School of Dentistry, Shiraz University of Medical Sciences, Shiraz, Iran.; 2 Student Research Committee, School of Dentistry, Shiraz University of Medical Sciences, Shiraz, Iran.

**Keywords:** Alkaline pH, Apical Plug, Calcium Enriched Mixture Cement, Fluid Filtration, Mineral Trioxide Aggregate, Microleakage, Sealing Ability

## Abstract

**Statement of the Problem:**

Alkaline pH can affect the physical and chemical properties and sealing ability of apical plug material. Calcium hydroxide is used as an intracanal medication to complete disinfection of root canals. It raises the pH of environment to alkaline value.

**Purpose:**

The aim of this *in vitro* study was to evaluate and compare the effect of alkaline pH on the sealing ability of calcium-enriched mixture (CEM) cement and mineral trioxide aggregate (MTA) apical plugs.

**Materials and Method:**

Seventy single-rooted human maxillary anterior teeth were randomly divided to two experimental groups for Angelus MTA and CEM cement (n=30) and two control groups (n=5). Each group was divided into two subgroups of 15 for neutral and alkaline pH, and 1 negative and 1 positive control groups of 5. The root canals were cleaned and shaped by using ProTaper rotary system (Dentsply Maillefer; Ballaigues, Switzerland) and the terminal 3mm of the roots were resected. Then, MTA and CEM cement were condensed in apical region with 3mm thickness. The samples were exposed to two environments with different pH values of 13 and 7.4. The leakage was assessed by using the fluid filtration technique at 1, 7, 14, 30 days intervals. Data were analyzed by the repeated measures MANOVA.

**Results:**

There was no statistically significant difference in the rate of microleakage between neutral and alkaline pH of CEM cement and MTA (*p*> 0.05). The sealing ability of MTA in an alkaline pH of 13 was significantly less than CEM cement in this pH (*p*< 0.05).

**Conclusion:**

An environment with alkaline pH had no adverse effect on the sealing ability of MTA and CEM cement used as apical plugs. CEM cement had better sealing ability in alkaline pH.

## Introduction

A non-vital immature tooth presents many complications to achieve an appropriate endodontic therapy.[1]Since the apex is extremely wide, no barrier exists to limit the obturation process of the root canal system.[1-3] A variety of materials including mineral trioxide aggregate (MTA) and calcium enriched mixture (CEM) cement have been used to create an apical stop in such cases. MTA has a good sealing ability, sets in the presence of blood and it is also biocompatible; all these properties make it a good candidate for apical plug.[4] However, it encompasses some drawbacks such as extended setting time, poor handling, and high cost.[5]On the other hand, CEM cement has good sealing ability and favorable biologic response, as well as greater flow, less film thickness [6-7] and shorter setting time (less than 1 hour) than MTA.[6-8]

The vast majority of non-vital teeth are infected, so the first phase of treatment is to disinfect the root canal system. A creamy mix of calcium hydroxide is used as an intracanal medication to complete disinfection of the root canals.[9-11] Calcium hydroxide kills bacteria, neutralizes the biologic activity of lipopolysaccharides and makes the necrotic tissues more susceptible to the solubility action of NaOCl.[10] Freshly-mixed calcium hydroxide produces an environment with alkaline pH.[12] It is impossible to remove all of the calcium hydroxide from the dentinal walls; so, the alkaline pH of this area can potentially affect the properties of freshly-applied apical plug materials.[10, 13] This pH conversion inhibits the setting reactions and increases the solubility of materials. Physical and chemical properties of materials affect their sealing ability.[14-16] Saghiri *et al.*[17] showed that the leakage of MTA in acidic pH occurred faster than the neutral pH. Lotfi *et al.*[11] concluded that alkaline pH affected the sealing ability of MTA. Hachmeister *et al.*[18] found that calcium hydroxide did not influence the sealing ability of gray MTA (GMTA). Stefopoulos *et al.*[19] reported that calcium hydroxide pretreatment adversely affected the sealing ability of MTA.

To the best of our knowledge, no investigation has evaluated the effect of alkaline pH on the sealing ability of CEM cement. Therefore, the aim of this *in vitro* study is to assess the effect of alkaline pH on the microleakage of MTA and CEM cement by employing fluid filtration technique. 

## Materials and Method

Seventy extracted human maxillary single-rooted teeth were collected. After extraction, the teeth were placed in 1% sodium hypochlorite for 48 hours to be disinfected and were then rinsed and stored in normal saline solution until used. The teeth were examined for cracks and calcified canals. Crowns were sectioned with a fissure bur (Maillefer; Ballaigues, Switzerland) in a high-speed handpiece and water spray to create a standardized root length of 15mm.

The canals were cleaned and shaped with ProTaper rotary system (DENTSPLY; Switzerland) according to the manufacturer’s instructions. The teeth were then randomly divided into two experimental groups (n=30) for Angelus MTA (Angelus; Londrina, Paraná, Brazil) and CEM cement (BioniqueDent; Tehran, Iran) and two control groups (n=5). Then each group was divided into two subgroups of 15 for neutral and alkaline pH. The root canals were cleaned and shaped by using ProTaper rotary system to F3 (Dentsply Maillefer; Ballaigues, Switzerland) according to the manufacturer’s protocol. During instrumentation, after each rotary file, two mL of 2.5% NaOCl was used. A fissure bur in a high-speed handpiece under copious water spray was used to cut 3 mm of the apical part of the roots perpendicular to the long axis. The apical region of the teeth was dilated with piezo up to size 3 to simulate open apex teeth. MTA and CEM cement were prepared according to the manufacturer’s instructions. The instrumented canals were dried with paper points (VDW; Munich, Germany), then the MTA and CEM cement were condensed to the apical end by using a plugger (Dentsply; Maillefer, Tulsa, USA) 3 and 4 with a rubber stop placed 3 mm shorter than the root canal length. In the experimental groups the thickness of apical barrier was confirmed by radiographs. The entire root surface was coated with two layers of nail varnish, except the area corresponding to the resected root-end surface. In the positive control group, no apical plug was used. In the negative control group, 3mm MTA apical plug was applied and the whole root surfaces and the surface including the end were coated by two layers of nail varnish.

Calcium hydroxide at pH value of 13 was used for preparing alkaline environment.[12] In alkaline pH experimental group, the teeth were placed in pieces of gauze smeared with calcium hydroxide for 3 days.[11] The gauze was replaced every day with fresh one to ensure a sufficient alkaline pH environment. The teeth in the other experimental group were placed in pieces of gauze, immersed in the synthetic tissue fluid with neutral pH (STF containing 1.7 g of KH2PO4, 11.8 g of Na2HPO4, 80.0 g of NaCl, and 2.0 g of KCl in 10 L H2O) for 3 days.[11]

The microleakage was evaluated by the fluid filtration technique employing a pressure of 20 cm H_2_O.[20] In this method, a device was prepared by attaching two micropipettes perpendicular to each other. The teeth were fixed at the end of a horizontal micropipette and 20 cm normal saline was inserted in the vertical micropipette, presenting the pressure of 20 cm H_2_O. Microleakage for each tooth was measured at 1, 7, 14, 30 day. Then, the bubble displacement of each interval (0-1, 1-7, 7-14, 14-30) was reported. The rate of microleakage (RML) of each interval was calculated and expressed in µL/min[21] (bubble displacement in each interval / period of interval).

In data analysis process, the repeated measures MANOVA test was employed to evaluate the change of sealing ability over time. The significant level was set at 0.05 for all tests. 

## Result

The bubble was not displaced in the micropipette in negative control group; while, the fluid flow was observed as soon as the pipette was opened in the positive control group.

All experimental groups demonstrated various amounts of microleakage (Table 1). 

**Table 1:** The mean (SD) of microleakage (µL/min/cm H_2_O×10³) for MTA and CEM cement as apical plugs in four time spans

	**pH level**	**Mean (SD)**				
		**Day 1**	**Day 7**	**Day 14**	**Day 30**	**N**
MTA	Alkaline	14.5(7)	3.6(2.8)	5.5(2.5)	4.1(1.4)	15
	Neutral	9.4(4.5)	2.3(1.2)	4.4(1.9)	3.3(1.3)	15
CEM	Alkaline	11.4(7.9)	2.1(0.8)	2.4(1.0)	2.4(0.9)	15
	Neutral	8.6(5.2)	1.8(1.3)	2.7(1.1)	2.1(0.9)	15

There was no statistically significant difference in RML between neutral and alkaline pH of CEM cement (*p*> 0.05). There was no statistically significant difference in RML between the neutral and alkaline pH of MTA (*p*> 0.05). This materials exhibited similar sealing ability in neutral pH. The RML of MTA in an alkaline pH of 13 was significantly greater than CEM cement in the same pH (*p*< 0.05).

**Figure 1:**
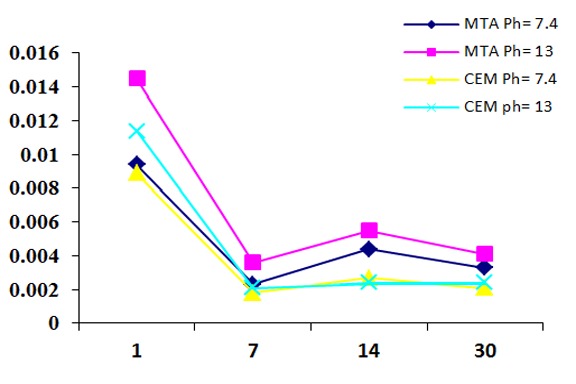
The mean microleakage of MTA and CEM cement as apical plugs with different pH levels and at different time intervals

The RML reduced over time and it was more detectable in the second interval (Figure 1).

## Discussion

This study compared the sealing ability of MTA and CEM cement as apical plugs in alkaline pH. Using calcium hydroxide as an intracanal medicament is effective in killing microbial flora and dissolving necrotic pulp tissue.[22-23] Freshly mixed calcium hydroxide creates an alkaline environment that reaches its maximum pH value after 8 hours (pH=13).[10] Several methods such as bacterial penetration, glucose leakage, dye penetration, and fluid filtration have been used to measure the microleakage around the materials.[25-28] Dye penetration and bacterial leakage are qualitative methods which may destroy the samples and do not allow evaluating the sealing ability over time. Passing the bacteria through the samples may show positive culture in bacteria leakage method. The reliability of any glucose study is questionable. Hydration of MTA results in calcium hydration which may react with glucose and affect the result of glucose leakage method. The fluid filtration is a quantitative and qualitative method that can record very small changes. This method does not destroy the samples; so it can be used to evaluate the sealing ability over time.

We used calcium hydroxide in this study to induce alkaline pH. A previous study showed that calcium hydroxide, as an intracanal drug, attached to the dentinal walls and directly interfered with the sealing ability of apical plug.[11] Therefore, we did not apply calcium hydroxide into the canals to eliminate this interference.

The current study found that alkaline pH had no adverse effect on the RML of MTA and CEM cement. This was similar to the results obtained by Bidar *et al.*[9] who found that the sealing ability of MTA did not change in the presence of calcium hydroxide.

 Hachmeister *et al.* detected that the residual calcium hydroxide in dentinal wall did not affect the sealing ability of MTA. In contrast, Bidar *et al.*[10] observed that medication with calcium hydroxide improved the marginal adaptation of MTA plug since calcium hydroxide produces calcium carbonate which decreases the permeability.

In another study, Bidar *et al*.[3] concluded that medication with calcium hydroxide did not influence the marginal adaptation of CEM cement.

Using calcium hydroxide would leave obstacle in dentinal wall and consequently affect the sealing ability of apical plugs. Thus, in order to eliminate the effect of mechanical obstacle in this study, only the tooth surface was exposed to the source of alkaline.

Concerning another result of our study, CEM cement in neutral pH group exhibited the lowest RML among the experimental materials; however, this difference was not significant. CEM cement has good handling characteristic and can be condensed easily.

According to the results of this study, the RML of MTA was significantly greater than the CEM cement in an alkaline pH. In such an environment, the calcium ions that are released from CEM cement and MTA react with phosphate to form hydroxyapatite which improves the sealing ability.[29] CEM cement has more calcium component and may make more hydroxyapatite.[29]

## Conclusion

Based on the results of this *in vitro* study, an alkaline pH environment had no adverse effect on the sealing ability of MTA and CEM cement as apical plugs. CEM cement had better sealing ability in alkaline pH. 

## References

[1] Hong ST, Bae KS, Baek SH, Kum KY, Lee W (2008). Microleakage of accelerated mineral trioxide aggregate and Portland cement in an in vitroapexification model. J Endod.

[2] Asgary S, Eghbal MJ, Parirokh M (2008). Sealing ability of a novel endodontic cement as a root-end filling material. J Biomed Mater Res A.

[3] Bidar M, Disfani R, Gharagozlo S, Rouhani A, Forghani M (2011). Effect of previous calcium hydroxide dressing on the sealing properties of the new endodontic cement apical barrier. Eur J Dent.

[4] Khalilak Z, Vali T, Danesh F, Vatanpour M (2012). The Effect of One-Step or Two-Step MTA Plug and Tooth Apical Width on Coronal Leakage in OpenApex Teeth. Iran Endod J.

[5] Asgary S, Eghbal MJ, Parirokh M, Ghoddusi J, Kheirieh S, Brink F (2009). Comparison of mineral trioxide aggregate's composition with Portland cements and a new endodontic cement. J Endod.

[6] Asgary S, Shahabi S, Jafarzadeh T, Amini S, Kheirieh S (2008). The properties of a new endodontic material. J Endod.

[7] Hasan Zarrabi M, Javidi M, Naderinasab M, Gharechahi M (2009). Comparative evaluation of antimicrobial activity of three cements: new endodontic cement (NEC), mineral trioxide aggregate (MTA) and Portland. J Oral Sci.

[8] Asgary S, Eghbal MJ, Parirokh M (2008). Sealing ability of a novel endodontic cement as a root-end filling material. J Biomed Mater Res A.

[9] Bidar M, Disfani R, Asgary S, Forghani M, Gharagozlo S, Rouhani A (2012). Effect of calcium hydroxide premedication on the marginal adaptation of calcium-enriched mixture cement apical plug. Dent Res J (Isfahan).

[10] Bidar M, Disfani R, Gharagozloo S, Khoynezhad S, Rouhani A (2010). Medication with calcium hydroxide improved marginal adaptation of mineral trioxide aggregateapical barrier. J Endod.

[11] Lotfi M, Vosoughhosseini S, Saghiri M, Zand V, Yavari HR, Kimyai S (2011). Effect of alkaline ph on sealing ability of white mineral trioxide aggregate. Med Oral Patol Oral Cir Bucal.

[12] Fulzele P, Baliga S, Thosar N, Pradhan D (2011). Evaluation of calcium ion, hydroxyl ion release and pH levels in various calcium hydroxide basedintracanal medicaments: An in vitro study. Contemp Clin Dent.

[13] Bidar M, Disfani R, Gharagozloo S, Akbari M, Rouhani A (2011). The Effect of Calcium Hydroxide on the Short and Long-Term Sealing Properties of MTA Apical Barrier. Iran Endod J.

[14] Torabinejad M, Hong CU, McDonald F, Pitt Ford TR (1995). Physical and chemical properties of a new root-end filling material. J Endod.

[15] Namazikhah MS, Nekoofar MH, Sheykhrezae MS, Salariyeh S, Hayes SJ, Bryant ST (2008). The effect of pH on surface hardness and microstructure of mineral trioxide aggregate. Int Endod J.

[16] Watts JD, Holt DM, Beeson TJ, Kirkpatrick TC, Rutledge RE (2007). Effects of pH and mixing agents on the temporal setting of tooth-colored and gray mineral trioxideaggregate. J Endod.

[17] Saghiri MA, Lotfi M, Saghiri AM, Vosoughhosseini S, Fatemi A, Shiezadeh V (2008). Effect of pH on sealing ability of white mineral trioxide aggregate as a root-end filling material. J Endod.

[18] Hachmeister DR, Schindler WG, Walker WA 3rd, Thomas DD (2002). The sealing ability and retention characteristics of mineral trioxide aggregate in a model of apexification. J Endod.

[19] Stefopoulos S, Tsatsas DV, Kerezoudis NP, Eliades G (2008). Comparative in vitro study of the sealing efficiency of white vs grey ProRoot mineral trioxide aggregate formulas as apical barriers. Dent Traumatol.

[20] Held D, Thron HL (1962). Studies on the circulation of bone. I. Tissue pressure of the marrow. Arch Sci Physiol (Paris).

[21] Inan U, Aydin C, Tunca YM, Basak F (2009). In vitro evaluation of matched-taper single-cone obturation with a fluid filtration method. J Can Dent Assoc.

[22] Sjögren U, Figdor D, Spångberg L, Sundqvist G (1991). The antimicrobial effect of calcium hydroxide as a short-term intracanal dressing. Int Endod J.

[23] Hasselgren G, Olsson B, Cvek M (1988). Effects of calcium hydroxide and sodium hypochlorite on the dissolution of necrotic porcine muscle tissue. J Endod.

[24] Saghiri MA, Lotfi M, Saghiri AM, Vosoughhosseini S, Aeinehchi M, Ranjkesh B (2009). Scanning electron micrograph and surface hardness of mineral trioxide aggregate in the presence of alkaline pH. J Endod.

[25] Moradi S, Disfani R, Ghazvini K, Lomee M (2013). Sealing Ability of Orthograde MTA and CEM Cement in Apically Resected Roots Using Bacterial Leakage Method. Iran Endod J.

[26] Vosoughhosseini S, Lotfi M, Shahmoradi K, Saghiri MA, Zand V, Mehdipour M (2011). Microleakage comparison of glass-ionomer and white mineral trioxide aggregate used as a coronal barrier in nonvital bleaching. Med Oral Patol Oral Cir Bucal.

[27] Veríssimo DM, do Vale MS (2006). Methodologies for assessment of apical and coronal leakage of endodontic filling materials: a critical review. J Oral Sci.

[28] Haghgoo R, Abbasi F (2013). Treatment of Furcal Perforation of Primary Molars with ProRoot MTA versus Root MTA: A Laboratory Study. Iran Endod J.

[29] Amini Ghazvini S, Abdo Tabrizi M, Kobarfard F, Akbarzadeh Baghban A, Asgary S (2009). Ion release and pH of a new endodontic cement, MTA and Portland cement. Iran Endod J.

